# The Unclear Role of the Physician on Social Media During the COVID-19 Pandemic. Comment on “Emergency Physician Twitter Use in the COVID-19 Pandemic as a Potential Predictor of Impending Surge: Retrospective Observational Study”

**DOI:** 10.2196/34870

**Published:** 2022-03-02

**Authors:** Spencer MacLeod, Nikhi Paul Singh, Carter Joseph Boyd

**Affiliations:** 1 School of Medicine The University of Alabama at Birmingham Birmingham, AL United States; 2 Hansjörg Wyss Department of Plastic Surgery NYU Langone New York City, NY United States

**Keywords:** COVID-19 pandemic, emergency medicine, disaster medicine, crisis standards of care, latent Dirichlet allocation, topic modeling, Twitter, sentiment analysis, surge capacity, physician wellness, social media, internet, infodemiology, COVID-19

We congratulate Margus and colleagues on their interesting study documenting emergency medicine physicians’ use of Twitter preceding surges in COVID-19 cases [1]. The correlations discovered between tweet count and hospital case numbers represent a unique instrument to assess epidemiologic trends related to the COVID-19 pandemic [1]. An additional subanalysis by geographic region may provide enhanced insight into the efficacy of social media utilization as a predictive tool for emergency medical resource allocation. We commend the authors for their extensive search criteria employed to accurately identify US emergency medical physicians; however, this study’s complex methodology highlights the lack of an official role and verification process for physicians on social media.

On Twitter, there is no specific criteria listed for verification of medical professionals. Instead, verification is based on notability criteria associated with representing a notable individual or brand [2]. These criteria exclude any physicians without a sufficiently large following from being verified on Twitter, making it more difficult for Twitter users to identify legitimate medical professionals lacking a large following. As health professionals and researchers are further encouraged to utilize social media for professional purposes, additional verification criteria specific to medical professionals may prove beneficial in the future [3].

In 2010, the American Medical Association issued guidelines regarding the ethical use policy to aid physicians in navigating social media [4]. However, the role of physicians on these online platforms has not been uniformly described. Physicians may interact with one another, and this is evident with Med Twitter, an open-source, decentralized forum for information sharing, medical education, and professional networking, as well as the increasing use of social media in residency recruitment [5]. The observed correlation by the authors of this study may be indicative of such communication by emergency medicine physicians. Continued use of such platforms across medicine may reveal additional relationships such as this predictive model. Perhaps a potential role may be to extend physicians’ professional impact in education and patient advocacy.

As time moves forward, the use of social media in medicine will continue to expand beyond prediction as will its potential pitfalls. Margus and colleagues’ article [1] brings up a positive prospect of social media in medicine, and it is important that physicians understand the current limitations of these innovative platforms. We believe that physicians need medicine-based verification for social media in addition to clearly established roles for physicians from national governing bodies.
